# Vitamin A status of healthy children in Manisa, Turkey

**DOI:** 10.1186/1475-2891-9-34

**Published:** 2010-09-01

**Authors:** Nermin Tansuğ, Muzaffer Polat, Selcan Çeşme, Fatma Taneli, Salih Gözmen, Özlem Tokuşoğlu, Dilek Yılmaz, Gönül Dinç

**Affiliations:** 1 Celal Bayar University, Faculty of Medicine, Department of Pediatrics, Manisa, Turkey; 2 Celal Bayar University, Faculty of Medicine, Department of Biochemistry, Manisa, Turkey; 3 Celal Bayar University, Akhisar MYO, Manisa, Turkey; 4 Celal Bayar University, Faculty of Medicine, Department of Public Health, Manisa, Turkey

## Abstract

**Background:**

Vitamin A deficiency is a major public health nutrition problem in the developing world. Even subclinical Vitamin A deficiency is associated with increased childhood mortality. Severe maternal vitamin A deficiency may cause increased mortality in the first months of life. There have been a limited number of studies regarding vitamin A status in Turkey. The aim of this study was to assess vitamin A status of healthy children in Manisa, Turkey.

**Methods:**

Vitamin A status of 100 healthy children aged 36-48 months is evaluated. The children were seen during routine examination. Serum retinol concentrations were measured by high-performance liquid chromatography. Duration of breast feeding, age solid foods introduced, use of supplementary vitamins, weight and height, and intake of specific groups of nutrients on a daily, weekly and monthly basis were collected from a questionnaire completed by the mothers. Height and weight z-scores were calculated according to national standards. Mothers of 20 of the 100 children were known to have normal serum and breast milk retinol concentrations. Children with normal serum retinol concentration were compared with the children with VAD. Student's t-test and Mann-Whitney test were used to compare independent variables. The Pearson correlation analysis test was used to test relation between numeric variables.

**Results:**

Mean retinol concentration was 0.98 ± 0.32 μmol/L in the whole study group. Serum retinol concentrations were normal (>0.70 μmol/L) in 89% of the children. When children with normal serum retinol concentrations were compared with those with retinol concentrations lower than 0.70 μmol/L, there was no difference in terms of age, gender, weight and height at the time of study, z-scores, birth weight, birth length, duration of breast feeding, time to begin solid food, rate of supplementary vitamin use, and rate of infections (P > 0.05). There was not any relation between vitamin A concentrations and weight and height at the time of study, z-scores, birth weight, birth length, duration of breast feeding, time to begin solid food, vitamin use, and frequency of intake of specific groups of nutrients (P > 0.05).

**Conclusions:**

This study showed that VAD is a moderate health problem in Manisa.

## Background

Vitamin A is a component of retinal pigments which has an important role in vision in dim light. It especially affects young children, among whom deficiency can cause xerophthalmia and lead to blindness, limit growth, weaken host defenses, exacerbate infection and increase the risk of death [1]. Vitamin A deficiency (VAD) can extend through school age and adolescent years into adulthood. VAD in women of reproductive age may increase morbidity and mortality during pregnancy and the early postpartum period [2,3]. Severe maternal VAD may also cause increased mortality in the first months of life [4,5].

VAD is a major public health nutrition problem in the developing world. According to the World Health Organization (WHO), serum retinol concentrations are classified as normal, marginal, and deficient, ≥0.70 μmol/L, 0.35-0.70 μmol/L, and <0.35 μmol/L, respectively [6]. Public health significance of VAD is categorized as mild, moderate, and severe. Degree of severity of VAD is mild if prevalence of preschool-age children or pregnant women with marginal or deficient serum plasma concentrations is 2-10%, moderate if prevalence is 10-20%, and severe if prevalence is ≥20% [7]. WHO defines two main groups from the viewpoint of VAD: Xerophthalmia and night blindness constitute a serious public health problem in the first group of countries, such as some of those in Africa and South-east Asia. In the second group of countries, clinical signs of VAD are rarely detected but marginal VAD has been determined in 10-30% of these populations and a continuous monitoring of vitamin A status is recommended. The Pan-American Health Organization (PAHO) considers VAD as a public health problem when 15% or more of the population display serum retinol concentrations of 0.70 μmol/L [8]. Among women, two retinol concentration cut-offs are used to estimate VAD and low to deficient vitamin A status, respectively, 0.70 μmol/L and 1.05 μmol/L [6,9]. A retinol concentration in breast milk less than 1.05 μmol/L is considered to be consistent with VAD in lactating women. Prevalence <10%, ≤10 to <25%, ≥25% of breast milk retinol 1.05 μmol/L are considered to indicate mild, moderate and severe VAD, respectively as a public health problem [9,10].

Normal concentration of vitamin A in breast milk is sufficient for normal health and development for infants. Since permeability of the placenta for liposoluble vitamins is limited and plasma retinol concentration of the newborn is low, amount of hepatic vitamin A store depends on breast milk vitamin A concentration. Breast milk of a well-nourished mother is sufficient for storage of vitamin A in the liver of the infant [1,9]. Other foods such as vegetables, fruit, egg, butter, and liver are sources of vitamin A. The risk of VAD is low for healthy children that consume a balanced diet. VAD may also be due to insufficient intestinal absorption secondary to chronic intestinal diseases or low dietary fat intake [1,7].

Diarrhea and severity of measles as well as deaths from these diseases had markedly decreased by the use of supportive vitamin A in developing countries where VAD is endemic. It has been reported that VAD is associated with increased incidence of respiratory and gastrointestinal infections, and that mortality for the age group of 1-5 years can be decreased by 35% by eradication of VAD [11-13].

There are a limited number of studies regarding vitamin A status in Turkey. Studies on sub clinical VAD among healthy children is even less. Majority of VAD studies are on VAD in children with malnutrition, diarrhea, measles and acute respiratory infections. On the study by WHO regression based estimate of VAD among preschool aged children inTurkey is moderate (12.4%) [7]. VAD is especially important for preschool children. In a previous study in Manisa province of Turkey, retinol concentrations in breast milk and mothers' serum were found within normal range [14]. We aimed to evaluate vitamin A status of healthy children in Manisa, Turkey, in this study.

## Methods

The study was conducted in Manisa, a city in the Aegean region of Turkey. A total of 100 healthy children were enrolled in the study. The children were seen at pediatrics outpatient clinics and health centers where they were followed for growth, development, and routine immunization. They were healthy children visiting the health centers routinely without any health problems. Ages of the children were ranging between 36 and 48 months. Demographic features, duration of breast feeding, age solid foods introduced, use of supplementary vitamins, weight and height, and intake of specific groups of nutrients on a daily, weekly and monthly basis and frequency of infections were collected from a questionnaire completed by the mothers. Height and weight standard deviation scores (z-scores) were calculated according to national standards [15]. Mothers of 20 of the 100 children were known to have normal serum and breast milk retinol concentrations. Blood specimens of each child were obtained and transported to the laboratory for detection of serum retinol concentrations. Samples were protected from light and heat during transportation. Serum retinol concentrations were measured by high-performance liquid chromatography (HPLC) (Chromsystems Diagnostics GmbH, München, Germany). Serum retinol concentrations were classified as normal, marginal and deficient according to WHO criteria [6].

Features, food habits, and measurements of the children with normal serum retinol concentration (non-VAD group) were compared with those of the children with VAD (VAD group).

Data were analyzed statistically by the statistical Package for Social Sciences (SPSS 11.0; SPSS Institute, Chicago, IL, USA) for Windows (Microsoft). Numerical values are given as mean ± standard deviation. Student's t-test and Mann-Whitney test were used to compare independent variables. The Pearson correlation analysis test was used to test relation between numeric variables. Significant relationships between variables were defined as P < 0.05.

The protocol for the study was approved by the ethical committee of Celal Bayar University Medical Faculty.

## Results

The age of the children ranged from 36 to 48 months, with a mean of 42.7 ± 5.3 months. Fifty-nine (59%) boys, forty-one (41%) girls were enrolled in the study. All of the children were within normal range in terms of weight and height. Mean duration of breast feeding was 13.6 months, and mean onset of supplementary food was 7.0 months. Supplementary vitamins were given to 85.2% of the children. All of the children were followed by a physician.

Mean serum retinol concentration was 0.98 ± 0.32 μmol/L. Serum retinol concentrations were normal in 89% of the children (1.06 ± 0.27 μmol/L), whereas low in 11% of them (0.47 ± 0.19 μmol/L). Serum retinol concentrations of two children were lower than 0.35 μmol/L.

The differences between the two groups in terms of age, gender, weight and height at the time of study, z-scores, birth weight, birth length, duration of breast feeding, time to begin solid food, percentage of supplementary vitamin use, and percentage of infections were not statistically significant (P > 0.05) (Table 1). There was not any relation between vitamin A concentrations and weight and height at the time of study, z-scores, birth weight, birth length, duration of breast feeding, time to begin solid food, vitamin use, and frequency of intake of specific groups of nutrients (P > 0.05).

**Table 1:** Differences between VAD and non-VAD groups.

	Non-VAD group (n = 89)	VAD group (n = 11)	P^†^
Age (mo.)*	38.8 ± 9.7	40.4 ± 9.6	0.58^†^
Gender	Boys 60%, Girls 40%	Boys 45%, Girls 55%	0.27^††^
Weight (kg)*	14.1 ± 2.7	13.7 ± 1.8	0.65^†^
Weight-for-age z-score	-0.4 ± 1.4	-0.6 ± 1.3	0.59^††^
Height (cm)*	90.0 ± 9.2	94.1 ± 4.1	0.19^†^
Height-for age z-score	-1.0 ± 1.5	-1.0 ± 1.5	0.78^††^
Birth weight (g)*	3267 ± 1307	3127 ± 734	0.58^†^
Birth length (cm)*	50.3 ± 2.7	49.5 ± 2.1	0.56^†^
Duration of breast feeding (mo.)*	14.2 ± 9.5	13.0 ± 7.2	0.80^††^
Age of onset of solid food (mo.)*	6.5 ± 3.1	7.5 ± 6.1	0.76^††^
Percentage of vitamin use	81.3%	85.7%	0.30^††^
Percentage of infections	51.4%	64.3%	0.30^††^

* Mean ± standard deviation, ^† ^Student's t test, ^† † ^Mann-Whitney test

## Discussion

VAD has received little attention since clinical signs such as xerophthalmia and night blindness are rarely seen. However, marginal deficiency may show negative effects on health and increase morbidity and mortality among preschool children even at subclinical concentrations [9]. VAD may worsen infection but vitamin A supplementation reduces the risk of death. VAD in women of reproductive age may increase morbidity and mortality during pregnancy and early postpartum period [2,3]. Severe maternal VAD may also lead to increased mortality in the infant in the first months of life [4]. In a recent study on breast milk and serum retinol concentrations in Manisa mean concentrations of retinol were found as 2.94 ± 0.31 and 1.29 ± 0.23 μmol/L in breast milk and serum, respectively, indicating that there is no VAD among lactating women in this region [14]. In another study on lactating women in İstanbul, retinol concentrations were marginal [16].

Mean serum retinol concentration was 0.98 ± 0.32 μmol/L, which is within normal limits, in this study. However, the rate of children with serum retinol concentration <0.70 μmol/L was 11%. According to data published by WHO, estimate of the prevalence of serum retinol concentration <0.70 μmol/L in preschool-age children 1995-2005 for Turkey is 12.4% which shows significance of VAD as a moderate public health problem [7].

There are a few studies regarding vitamin A status of children in Turkey. Hatun and Teziç, in their study with 80 children at the ages of 9-17 months in Ankara, reported that 30% had serum retinol concentrations <0.70 μmol/L whereas 1.3% had serum retinol concentrations <0.35 μmol/L. They concluded that VAD is an important public health problem in that population [17]. In the study by Açkurt et al, rate of marginal and deficient retinol concentrations among healthy children at the ages 7-17 years were 9.3% and 2.3%, respectively [18]. VAD is not an important concern according to the latter study but the age group of the study population was higher than the others. Kurugöl et al reported serum retinol concentrations <0.70 μmol/L in 15.6% of 6-59 month-old children in İzmir, which is a neighboring city of Manisa [19]. In another study in the same city serum retinol concentrations of 62 children who were admitted in the hospital with pneumonia but without malnutrition or chronic diseases were evaluated. Serum retinol concentrations of 6.5% of the children were 0.35-0.70 μmol/L, but none of the children had serum retinol concentration <0.35 μmol/L [20]. Tıraş et al, in their study in Ankara, reported that 35% of a group of 20 children with a mean age of 11.1 months had serum retinol concentrations <0.35 μmol/L [21].

It has been reported that there was not any relation between feeding habits and retinol concentrations in the previous study on breast milk and serum retinol concentrations in lactating women in Manisa. Similarly we did not find any relation between feeding habits of the children and their serum retinol concentrations. This may be due to insufficient consumption of vitamin A rich foods in our region. Kurugöl et al have reported a positive relation between feeding habits and serum retinol concentrations [19].

In most of the studies serum retinol concentrations were measured to determine vitamin A status [7]. However measuring serum retinol concentration has some disadvantages. Serum retinol concentration reflects the body vitamin A status only when the liver retinol stores are seriously depleted and inflammation affects serum retinol concentration. Indirect methods such as the relative dose response and modified relative dose response tests are more sensitive. The tracer dilution technique is the only method that provides a quantitative estimate of total body vitamin A pool size. However these tests can not be used widely used since they are expensive. Direct measurement of liver reserves of vitamin A status through biopsy is rarely an option and therefore has no utility globally [22]. Thus, serum retinol concentration continues to be widely used to assess vitamin A status. Serum retinol concentration is used in the present study and since the children in the study group were healthy and had no complaints or no sign of illness, inflammation parameters were not measured.

Although the number of children enrolled was small, the percentage of preschool-age children with sub clinical VAD in this study suggests that monitoring of vitamin A status is needed in Manisa province. Considering that serum retinol concentrations are measured low as a consequence of serious depletion of body stores, these children may present with VAD disorders in case of severe infections that cause a decrease in vitamin A intake.

## Conclusions

VAD is a moderate public health problem in Manisa. In order to determine VAD category in Turkey, multicenter studies with larger groups should be carried out. Supplementary vitamin A may be beneficial in high risk groups such as malnourished infants and children with chronic infections. Families should be informed about improving feeding habits to increase use of vitamin A rich foods. Studies with larger group of children and with more parameters may be useful.

## Abbreviations

VAD: Vitamin A deficiency; WHO: World Health Organization; PAHO: Pan-American Health Organization; HPLC: High-performance liquid chromatography.

## Competing interests

The authors declare that they have no competing interests.

## Authors' contributions

NT: Participated in the conception, design, analysis and interpretation of data, manuscript preparation and revision. MP participated in the design of the study. SÇ carried out acquisition of the data and helped to draft the manuscript. FT carried out the HPLC studies. SG, ÖT, DY participated in the design of the study. GD performed the statistical analysis. All authors read and approved the final manuscript.
